# Virginia State Dental Association—Sixth Annual Session

**Published:** 1876-02

**Authors:** 


AMERICAN JOURNAL

OF

DENTAL SCIENCE.

Vol. IX. THIRD SERIES—FEBRUARY, 1876. No. 10.

### ARTICLE I.

### *Virginia State Dental Association—Sixth Annual Session.*

(*Reported Expressly for this Journal.*)

FIRST DAY.—MORNING.

The Sixth annual meeting of the Virginia State Dental Association was held at Association Hall, Richmond, on Tuesday, December 14th, 1875, at noon. Dr. W. W. H. Thackston, President, in the chair, and Dr Geo. F. Keesee, Secretary.

The Chair announced the presence of Dr. R. Finley Hunt, of Washington city, and invited him to a privileged seat in the hall.

The reading of the proceedings of the last session of the Dental Association was dispensed with, on motion of Dr. W. L. Burton.

Drs. Arthur Wendlinger and Frank L. Harris were elected members of the Association.

On motion of Dr. Burton, the Association took a recess until 4-30 P. M.

*Afternoon Session.*

The Association resumed its session at 4 P. M.

Professor Hodgkin, of the Baltimore Dental College, was introduced by the Chair to the Association, and invited to a privileged seat.

Dr. G. H. Chewing was, under a suspension of the rules, elected a member of the Association.

Dr. Cowardin, from the executive committee, reported that he had discharged the duties devolving upon that committee—procured a hall and made other arrangements for the session of the Convention. The report was adopted.

The Chair announced that he had addressed letters to the Presidents of the several railroads coming into this city requesting an abatement of railroad fare to the delegates, &c. All the roads had declined such abatement except the Atlantic, Mississippi and Ohio railroad.

On motion of Dr. Keesee, Dr. R. Finley Hunt, of Washington, was elected a corresponding member of the Association.

Dr. Hunt said he felt proud of the compliment, and would do all in his power to discharge the duties and responsibilities.

On motion of Dr. Thompson, Professor Hodgkin was elected an honorary member of the Association.

Dr. Keesee read a letter from Dr. Judson B. Wood, who s now in Baltimore, regretting that he was unable to be present at this session of the Association.

On motion of Dr. Steele the letter was read and ordered to be spread upon the record, and thanks returned to Dr. Wood.

Letters from Dr. N. M. Burkholder, of Harrisonburg, and Dr. John W. Scribner, of Charlottesville, expressing regret at not being able to be present, were read.

The letters were ordered to be spread upon the journal of the Association.

Dr. Steele made a personal explanation relative to the publication of the proceedings of the last session of the Dental Association.

The President next read his annual address to the Association.

[This excellent address will appear in the March No. of this JOURNAL.]

On motion of Dr. Thompson a vote of thanks was tendered the President for his able address, and a copy of his address was requested for publication in the *Journal.*

On motion of Dr. Cowardin it was ordered that Dr. Johnson, of Staunton, be allowed further time to report on Pathology and Surgery, he not having received information of his appointment as chairman of that committee.

Dr. Cowardin offered a chair and instructions for clinical operations, which were fixed from 8½ to 10½ A. M., daily during the sessions of the Association.

On motion the hours for the sessions of the Convention were fixed at from 10½ to 2 P. M., and from 4 to 7 P. M.

The Chair announced that the committee on Clinics for to-day would consist of Professor Hodgkin, Drs. T. L. Sydnor and F. L. Harris.

On motion the Association adjourned until 10½ o'clock Wednesday.

### SECOND DAY'S PROCEEDINGS.

The report of the Committee on Dental Pathology and Surgery was called for.

Dr. Johnston, of Staunton, Chairman of that committee, stated that not being aware of his appointment he had not prepared a report.

The subject was discussed by Dr. R. F. Hunt, of Washington, D. C. The subjects of Etiology and Pathology he said overlapped, and there was a difficulty in defining the limits of each, so that our discussion of the one would naturally embrace in part the other. The causation of decay in teeth is caused in a great measure by our general habits of living, our artificial life, our diet, &c., planting in us the seed of decay, in which our teeth share. He alluded with approval to the theory of Dr. John Allen, of N. Y., that the great loss of teeth by Americans was due to the abstraction of the mineral elements from our flour—the remedy being to use unbolted flour more largely. He brought up and discussed the action of the Washington and Baltimore Dental Associations, which bodies had put on foot an organized plan for obtaining statistics concerning the teeth of all the nations of the earth, in which they had endeavored to enlist the assistance of the U. S. Government, and hoped to obtain substantial results that would greatly advantage the profession, and also the public. He incidentally alluded to the fact that the Government had appointed dentists to the Navy yard at Annapolis and West Point, to rank as assistant surgeons.

Dr. W. L. Burton, of Richmond, spoke approvingly of special modes of diet, as influencing the development of the teeth, and alluded to the "Banting" cure for obesity in illustration of the correctness of his views. He had no doubt that a systematic diet would go far towards the formation of good teeth. He stated that his observation had led him to conclude that the teeth of negroes were now much inferior in quality and resistance to decaying influences than in former years.

Dr. Frank Harris, of Harrisonburg, Va., thought that the acidity of the saliva of females in pregnancy, was the cause of their decay at that period, and thought that the administration of the lacto-phosphates of lime, and other preparations of this character would be of benefit. He felt sure that the rising generations would see the result and be benefitted by our efforts; and that the time must come when the efforts of science would result in an improved condition of the teeth of our posterity. He considered the lack of the phosphates of lime in the system, the great cause of the defective teeth of the present generation.

Dr. T. L. Sydnor thought that decay was not the result of chemical action, but that causes back of this were in operation.

Dr. J. F. Thompson contrasted the modes of living at present, with those of past years. He agreed with the other speakers, that the teeth of the negro population had greatly deteriorated. A few years ago the calls for extracting teeth and performing other operations for them, were few compared with now. He thought the Scotch nation owed in part their good teeth to their oat meal diet.

Dr. Michard, of Richmond, called attention to the fact that the teeth of mulatoes were of very inferior quality. He thought also that the teeth of farmers were deteriorating.

Dr. S. H. Henkel, of Staunton, Va., thought that a correct pathology was the groundwork of a correct system of treatment of dental diseases. We should consider the subject of climate in endeavoring to get at the true course of dental caries. Crossing the blood of different races was a direct violation of the laws of nature, and all such violations were sure to be punished. Decay was the inevitable result of a violation of any of nature's laws. The iniquity of the parents was visited on the children. As to the cause of irregularity of development of teeth, he confessed himself sadly puzzled to account for some of the cases that come under his notice; no theory so far as he had seen, satisfactorily accounted for these deviations from a normal type. He gave a case in point, in which great irregularity was seen in the arrangement of the teeth of a young patient of his, the teeth of whose ancestry for three generations back were perfect in form and arrangement. He thought a necessity was upon us to investigate this subject, and give it our best efforts.

Dr. W. L. Burton, of Richmond, enquired if benefit might not result from the internal use of lime matter in cases of pregnancy, accompanied with softening cf the teeth. He was in hopes that statistics from the valley of Virginia and other lime stone regions would throw light on this obscure subject.

The President, Dr. Thackston, was gratified at the dis cussion and thought those suggestions and expressions of opinions, would stimulate to further research in this direction.

He gave a synopsis of the treatment of a case of alveolar abscess, involving extraction and replacement, which possessed he thought sufficient interest to deserve recital.

Dr. Henkle reported two successful cases of a somewhat similar nature. He thought that this operation had been removed from the list of impossibilities and even of improbabilities, to that of reasonable certainty. One of the cases in which he had removed the lower left bicuspid, and replaced after treatment, was operated on three and a half years ago and was still a success. The other was a lower molar, and was, though not of so long standing, still doing well. Age of the first patient 18 years.

Prof. Hodgkin. This is a subject of absorbing interest to the dentist, and I am glad that it is being discussed. Still I must dissent in part from the views expressed, or at least I do not fully endorse all that has been said. No one cause was in operation to produce the grave results that was seen so manifest in the loss of the teeth of the present generation, but the roots of this trouble are deep, far reaching and multitudinous. There are so many factors entering into this calculation ; causes remote and seemingly trivial are associated with those that are plain and obvious, to bring about the pathological conditions we see daily.

My friend Dr. Thompson, has quoted the authority of Dr. John Allen, a man whose opinion is certainly entitled to great weight, as saying that the bolting of the mineral elements from our staff of life, was the cause of the deterioration of the teeth of this generation. But in the face of this I put the opposite fact that the Irish race, who subsist mainly on the potato, an article of food containing little else save starch, have good teeth. We must seek elsewhere in part for the cause of this evil. May we not find much in the artificial life of the people to bring about the results in question ? Intra-uterine life is the time when the causes that produce bad teeth are most active. Children are begotten in debility, are born of feeble and ænemic mothers, are raised like hot-house plants with nothing natural in their surroundings and it is no wonder that their teeth share in their general feebleness of structure, which their bodies may outgrow, but which their teeth cannot. I cannot make up my mind to accept some of the theories broached, of supplying the system with such food as the teeth require. I did not believe and cannot until further evidence is produced, that the administration of lime salts can arrest caries. Teeth are not attacked from within, but from without, and no one has ever proved that they are robbed of their lime salts by its abstraction from the dentine through the agency of the pulp. It is a very pretty theory that we can feed the teeth from within, (some seem to have an idea that the enamel would or could take up lime salts from without,) but the facts stare us in the face that there are people who live on food almost wholly carbonaceous, with well developed, good solid teeth, (the Esquimaux, for example.) The solution of the difficulty lies far back of this. There is an abundance of lime salts in the ordinary diet of any child to form all the teeth it will have. Animal food is rich in phosphates, so are the legumens, beans, peas, &c. The fact that the Scotch had fine teeth could not be due solely to their living on oat meal, which, though a food rich in phosphates is yet not so rich as wheat meal.

I have under close observation two children, the parents of which having teeth of feeble structure themselves and are in feeble health, hoped to find in this doctrine of Dr. Allen, the means of constructing good teeth for their offspring.

The attention was first called to the subject when the first born child was only a few months old, and I suppose that no more faithful bran-eaters ever ate Graham mush. The mother was nursing her infant and she subsisted largely on bread made of unbolted wheat flour. So soon as the babe was of age to take other food than its mother's milk, it was fed upon the same diet, and it was faithfully kept up. In the course of time, the temporary teeth were erupted, only to show themselves soft, chalky and easily destroyed. Meantime the mother became again pregnant, and still firm in the resolution to give the theory a faithful trial, she during this term still used the unbolted flour, which had at least the effect to obviate the habitual constipation with which most pregnant females suffer. The same care in nursing the second child was observed, and the same diet used from the time it first took other food than milk, viz: unbolted wheat meal. The first child is now nearly five years of age, with teeth so soft and frail as to render it a matter of exceeding doubt if any of them will be lost in the natural way, in spite of the utmost care which its father, a dentist, has been able to give them. The other child now two and a half years old, has teeth but little if any better—soft, chalky, already extensively attacked by caries.

It is not one swallow, however, that makes a summer. I have seen a child of five years, whose teeth, developed under circumstances similar to those just related, are of most excellent quality. It should be stated that this last mentioned child, with teeth of exceptionable hardness and perfection of form, was raised not only on bran bread, but if the statement of his parents was to be accepted as evidence, had excluded from his diet sugar, salt, meats of all kinds, condiments, tea, coffee, &c. Whether this exclusion influenced the development of his teeth or not I cannot say.

A theory has lately been broached that the defective teeth of children is owing to the meagerness of their diet; that instead of being sent snpperless to bed, or with only a slight meal at that hour, that they should be well fed, on the assumption that digestion goes on more perfectly during sleep. The ground for this conclusion being that experiments on dogs fed and allowed to sleep, and dogs fed and hunted vigorously, proved that the quiet animals digested their food perfectly, while in those that were actively exercised the food was not digested. A comparison, however, between the children who are allowed to stuff themselves at night, and those whose diet was restricted, would show I think no good reason for allowing the stuffing practice to prevail.

An informal discussion here arose between members, as to the cause of sensitiveness in dentine, some attributing to the contents of the dentinal tubuli nerve functions, and considering them as true prolongations of the pulp.

Prof. Hodgkin. I am not prepared to accept the theory that the contents of the tubuli are nerve tissue. That they do convey sensation is a fact, but that they are ramifications of the pulp does, it seems to me, contradict the testimony of my eyes. Break a tooth open and what do you see? the pulp not closely adherent to the walls, as would surely be the case if thousands on thousands of fibres passed out into the dentine, but it is so loosely attached that it literally falls out, as the kernel of a nut falls out when the shell is broken.

Dr. Thackston. I must take issue with Prof. Hodgkin in this matter. I think the researches of Tomes and others conclusively prove that the contents of the tubules are continuous with the pulp, and have even been seen repeatedly, stretching out, partly pulled out of their respective lodgments, under the microscope. That the pulp is found as Prof. Hodgkin asserts is true, but he must remember that he is examining an extracted tooth, and the force used in removing it and severing the pulp at the foramen, might eat the pulp from the walls, and so produce the result seen, which might not be normal.

Dr. J. Hall Moore. I would like to ask Prof. Hodgkin if he does not find it difficult to remove a pulp not devitalized from the pulp chamber, and is not the fact that it adheres closely to the walls, requiring to be torn out in fragments, prove that it has a closer connection than he is willing to admit? I grant that pulps for some little time devitalized do come away easily, but those that are forcibly extirpated seem to cling with great tenacity to the walls of the pulp chamber, showing an attachment of some sort.

Prof. Hodgkin. The pulp is rather easily torn, and the aperture through which we obtain access to it is small, and the instruments not such as readily grasp it. These seem to me sufficient causes for the results spoken of by Dr. Moore, and so far as Mr. Tomes is concerned, with all due respect for such high authority, I must say, that I prefer my eyes and their evidence to his. I am aware that great reliance is placed on the evidence of the microscope, and the conclusions that are drawn from observations with that instrument are usually deemed infallible. But sir, when the microscopists cannot agree among themselves as to the form of blood discs, when they disagree as to the results of so many of their researches, what am I to infer—naturally that the instrument is not an infallible test of all things at least. I recollect a discussion that was going on in a late number of the *Popular Science Monthly*, in which it was shown conclusively that the very test relied on by the microscopists as evidence of certain powers of their certain instruments—a test accepted by all as fixed and conclusive—was no test at all, and that the best observers saw the same things in totally different shapes and forms.

Dr. Henkel. Man is highest production of his Creator, and we can neither violate his laws with impunity or set them aside. That we are constantly violating these laws is palpable, and it is no wonder that we suffer the penalty.

Dr. J. Hall Moore. I desire to call the attention of this Association to the unfavorable results obtained at my hands from the use of salicylic acid. I wish to warn my professional brethren not to trust this new agent, as it is in my opinion unreliable. In the treatment of teeth with dead pulps, I have not found it to yield the good results claimed for it, and I shall hereafter depend on carbolic acid, creosote, or nitrate of silver. I think salicylic acid a very efficient deodorizer and nothing more.

After further discussion the Association adjourned.

### Second Day.—*Afternoon Session.*

The subject of Dental Therapeutics was discussed. Dr. Henkle from the committee on this subject made a report.

Dr. W. L. Burton, approved of carbolic acid and the ordinary treatment of alveolar abscess. He had used salicylic acid, but could not report with what success as yet, as the treatment was recent. He noticed that it acted on the mucous membrane much as an escharotic.

Dr. Cowardin has found creosote and iodine superior to carbolic acid. He used iodine on the gum and in the root in three cases of abscess with success. He had been uniformly successful in his treatment of these cases.

Dr. Burton thought that in those cases where there was no fistulous opening through the gum, that creosote occasionally gave trouble. He had found the discharge of pus increased by its use.

Dr. Hunt spoke of injury to the tooth and pulp from the presence of amalgam. He had noticed that the dentine around and under an amalgam filling, was often blackened and hardened. The same result was seen under a tin filling. The sensitiveness of the dentine was much reduced. Now might we not here find a clue to a remedy for sensitiveness of dentine? What is this agent that hardens and deadens the dentine? is it the oxide of tin? This is a subject worthy of our investigation, and he hoped members of the Association would act on the hint he had given. He considered creosote invaluable, and iodine only second to it as agents for the cure of abscess.

Dr. Chewing thought tin a better filling than amalgam, and hoped to see it used more extensively.

Dr. Hunt wished to set himself right on the record. He did not wish the impression to be produced that he thought teeth were habitually killed by the use of amalgam. It (amalgam) was a good thing. Gold itself sometimes causes the death of the pulp. He had had sad experience with arsenic, and would warn members from using it for the purpose of obtunding sensitive dentine. He found he had killed in his day lots of teeth with it. There was a plain path right through the tubuli to the pulp, and it would certainly pass through.

Dr. Henkle confirmed the views of Dr. Hunt, on the subject of the use of arsenic

Dr. Burton spoke of the use of Guillois' cement as an agent for obtunding sensitive dentine, and also for capping nerves. He had great success with it.

Dr. Hunt asked if Guillois' cement was an oxychloride of zinc? He thought there was a difference between it and the ordinary os-artificial preparations.

Dr. Burton said that he was sure that the two were dissimilar. He had seen that chloride of zinc caused much more pain than the other. He spoke of a case in which the pulp was removed from a pulp chamber, but was alive in the roots of an upper molar, and he left it in these roots as it was healthy, and capped over.

Prof. Hodgkin expressed surprise that at this day any one thought it necessary to call attention to the injurious action of arsenic.

Dr. Thackston called attention to an article once popular, then disused and once again revised—the oil of cloves. He thought it worth a fair trial.

The subject of Dental Pathology was then passed.

The report of the committee on Physiology was called for. Dr. Burton, the chairman, had no report. Subject passe l.

Histology and Microscopy called. Dr. J. Hall Moore had no report. He had no microscope, and could not make investigations. Adjourned.

TO BE CONTINUED.

